# A frame orientation optimisation method for consistent interpretation of kinematic signals

**DOI:** 10.1038/s41598-023-36625-z

**Published:** 2023-06-14

**Authors:** Ariana Ortigas Vásquez, William R. Taylor, Allan Maas, Matthias Woiczinski, Thomas M. Grupp, Adrian Sauer

**Affiliations:** 1 Research and Development, Aesculap AG, Tuttlingen, Germany; 2 Department of Orthopaedic and Trauma Surgery, Musculoskeletal University Center Munich (MUM), Campus Grosshadern, Ludwig Maximilians University Munich, Munich, Germany; 3 Laboratory for Movement Biomechanics, ETH Zurich, Zurich, Switzerland

**Keywords:** Biomedical engineering, Musculoskeletal system, Orthopaedics

## Abstract

In clinical movement biomechanics, kinematic data are often depicted as waveforms (i.e. signals), characterising the motion of articulating joints. Clinically meaningful interpretations of the underlying joint kinematics, however, require an objective understanding of whether two different kinematic signals actually represent two different underlying physical movement patterns of the joint or not. Previously, the accuracy of IMU-based knee joint angles was assessed using a six-degrees-of-freedom joint simulator guided by fluoroscopy-based signals. Despite implementation of sensor-to-segment corrections, observed errors were clearly indicative of cross-talk, and thus inconsistent reference frame orientations. Here, we address these limitations by exploring how minimisation of dedicated cost functions can harmonise differences in frame orientations, ultimately facilitating consistent interpretation of articulating joint kinematic signals. In this study, we present and investigate a frame orientation optimisation method (FOOM) that aligns reference frames and corrects for cross-talk errors, hence yielding a consistent interpretation of the underlying movement patterns. By executing optimised rotational sequences, thus producing angular corrections around each axis, we enable a reproducible frame definition and hence an approach for reliable comparison of kinematic data. Using this approach, root-mean-square errors between the previously collected (1) IMU-based data using functional joint axes, and (2) simulated fluoroscopy-based data relying on geometrical axes were almost entirely eliminated from an initial range of 0.7°–5.1° to a mere 0.1°–0.8°. Our results confirm that different local segment frames can yield different kinematic patterns, despite following the same rotation convention, and that appropriate alignment of reference frame orientation can successfully enable consistent kinematic interpretation.

## Introduction

The development of an affordable and mobile alternative to current state-of-the-art gait analysis systems (such as marker-based or markerless optical motion capture, and static or moving videofluoroscopy^1,2^) could allow experts to better incorporate objective assessment of patient function into daily clinical practice. Most notably, the accurate estimation of rotational knee kinematics from inertial measurement units (IMUs) has received considerable attention in recent years^3^. Assessing these technologies to establish which approaches are able to provide a correct interpretation of the underlying kinematics requires their accuracy to be evaluated.

In clinical movement biomechanics, kinematics can be plotted to characterise joint motion over time. Two (or more) of these signals are then often compared to determine whether significant differences in kinematic patterns are associated with, for example, different pathologies^4,5^, disease stages^6,7^, treatment strategies^8,9^, or measurement systems^10–12^. In previous work, inertial-based knee kinematic estimates were compared against ground truth data generated by a calibrated joint simulator, using an analytical approach that allowed flexibility in the orientation of the sensor placement on the joint segments^13^. By assuming that the simulator output represented a true and correct measurement of the joint kinematics, the accuracy of the IMU estimates was quantified by calculating the root-mean-square error (RMSE) and maximum absolute error. This comparison relied on the fundamental assumption that the kinematics originating from each source (IMU-based system and robotic joint simulator) could be directly compared and would ideally be identical, therefore producing a consistent interpretation of the underlying movement patterns. This previous work demonstrated that accuracies (RMSEs) in the ranges of 0.4°–1.2°, 0.3°–5.5° and 0.7°–7.5° for angles in the sagittal, frontal, and transverse planes respectively, could be achieved using a model-based method to derive rotational knee kinematics from IMU data^13^. However, given that the testing scenario did not include deviations due to soft tissue movement, the observed level of errors was thought to be insufficient to reliably support clinical decision-making. Since the underlying segment kinematics were fundamentally the same, it is entirely plausible that the observed errors result from differences in the reference frames. Although this concept is well appreciated in the field of movement science, the practical implementation of consistent reference frames, especially when using IMU technology, has remained almost impossible.

When presenting any set of kinematic data, the associated coordinate frames of the individual joint segments *must* be clearly defined. In general, each body segment is assigned a three-dimensional (3D) Cartesian coordinate system. For rotational kinematics, this coordinate frame definition should, at a minimum, describe how the exact orientation of each of the three axes is determined. In cases where translational data is also included, each segment’s coordinate frame should be complemented by an explanation of how the location of the frame’s origin is established. Critical problems arise when these core requirements fail to be met. This failure commonly stems from a lack of consensus in the understanding and interpretation of approaches^14^. The popular Grood and Suntay Joint Coordinate System (JCS) was initially presented as non-orthogonal and sequence independent^15^. In practice, use of the JCS to describe e.g. knee kinematics is equivalent to individually assigning the femur and tibia a right-handed 3D Cartesian frame, and calculating a Cardan sequence describing the orientation of the distal (tibial) frame relative to the proximal (femoral) frame. This transformation follows an intrinsic sequence of rotations analogous to what would be clinically construed as: (1) flexion/extension, (2) adduction/abduction, and (3) internal/external rotation. In fact, the mathematical proof substantiating this interpretation has been previously reported, demonstrating the JCS to be both sequence dependent and orthogonal^16,17^. These controversies have contributed to confusion surrounding the definition of joint reference frames. Statements such as “kinematics were calculated according to Grood and Suntay” have unfortunately become commonplace, but insufficient if not accompanied by unambiguous details. A multitude of variations in the definition of tibiofemoral frames alone can be observed in the literature, even among those explicitly citing Grood and Suntay^14,18^.

Movement scientists attempt to standardise measurements of motion by using reliable anatomical or functional landmarks. However, different approaches used to analyse consistent kinematics have shown to result in considerably different interpretations of the joint motion^14,19^. In fact, the authors of this work strongly suggest that cross-talk between the different analysis approaches produces the large errors observed, and are therefore the underlying source of the very different interpretations. Cross-talk itself is a phenomenon whereby the alignment of the local segment coordinate system allows the rotation around one axis to be mixed-up with rotations around the others (see Supplementary Fig. S1 for an illustration of this effect). As a result, the measured rotations around each axis heavily depend upon the orientation of the chosen coordinate systems and to date assessment of motion patterns generally remains insufficiently reliable to support clinical decision-making.

To mitigate this problem, previous studies have explored methods of post-processing kinematic data to eliminate cross-talk. Woltring considered reducing cross-talk by transforming local segment frames so that ab/adduction or both ab/adduction and int/external rotation were zeroed at maximum flexion^20^. From a clinical perspective, however, the inherent assumption that there must be no ab/adduction and/or int/external rotation at maximum knee flexion of a gait cycle is questionable. Baker and co-workers, on the other hand, minimised ab/adduction variance, under the assumption that medio-lateral stability could be approximated as a hinge^21^, and therefore any variation in marker-based ab/adduction measurements was a likely result of thigh marker misplacement^22^. Rivest addressed cross-talk by transforming local segment frames to minimise the quadratic variation of ab/adduction and int/external rotation, but only applied the transformations in a weighted manner to minimise between-subject variability^23^. Cross-talk reduction thus depended on the assessed subject population, and post hoc inclusion of any additional participants would require complete recalculation of all subjects’ kinematics. Furthermore, for a given subject’s trial, the analysis could lead to different kinematic values, simply by being processed as part of different cohorts.

More recently, Baudet and co-workers proposed a cross-talk correction method based on principal component analysis, whereby variables were linearly transformed to eliminate correlations and minimise ab/adduction variance^24^. The authors concluded that the “correction method eliminated the presence of knee joint angle cross-talk, as proved by mean r^2^ values close to 0 for the left and right side after correction”, where r was the correlation coefficient between flexion/extension and ab/adduction. While r can be used as a descriptive measure of the *linear* association between two variables^25^, sensitivity analyses have shown that the relationship between flexion/extension and cross-talk artefact out of the sagittal plane is not linear (Supplementary Fig. S1)^26^. By extension, the assumption that cross-talk is equivalent to the linear relationship between flexion/extension and ab/adduction (rather than ab/adduction *error*) is an inherently misleading oversimplification. Furthermore, even if this linearity approximation were justified by limiting analyses to a confined range of knee flexion (i.e. where the relationship could be considered to be linear), r^2^ has repeatedly been criticised for being misinterpreted and confused with R^2^ (the coefficient of determination)^27,28^. While numerically equivalent to r^2^ under specific conditions, the idea that an R^2^ value close to zero indicates that two variables are not related is incorrect. Reducing r^2^ (or R^2^) between flexion/extension and ab/adduction to zero is therefore not the same as eliminating cross-talk. Moreover, Baudet and co-workers also justified the minimisation of ab/adduction variance by suggesting that previous cadaveric studies had managed to measure the knee’s physiological range of motion (ROM), and this range was small in ab/adduction^29^. This argument ignored the fact that, of all three reported rotations, ab/adduction was likely affected by the largest errors. Importantly, these ab/adduction measurements must have been associated with a set of local segment frames that were themselves susceptible to cross-talk, making it impossible to assume they unequivocally represent the joint’s true physiological ROMs.

A new perspective on knee kinematics is therefore critically necessary; one that recognises that any musculoskeletal kinematic measurement is the result of a series of *choices* designed to help us empirically characterise the highly complex 3D and time-dependent motion of an articulating joint. The fact that some of these choices may be more intuitive than others does not imply they are inherently *correct* or *incorrect*. Without the ability to reference values of some known true physiological joint motion, the following question arises: Given sets of kinematic data, can a consistent interpretation of the underlying movement patterns be achieved, independent of the analysis approach used? Answering this question is of critical importance in order to allow a standardised understanding of whether joint movement patterns are fundamentally similar or different, and therefore reliably support clinical decision-making. In this study, we directly address this challenging question by considering a Frame Orientation Optimisation Method (FOOM) that aligns reference frame orientations and corrects for cross-talk errors between kinematic signals derived using two different analysis approaches, with the goal to produce a consistent interpretation of the underlying articulating joint movement patterns.

## Methods

In this study, we present and investigate a Frame Orientation Optimisation Method that ensures consistent reference frame orientations and corrects for cross-talk errors in kinematic datasets. By executing rotational sequences to minimise cross-talk error between segment frames, we target a reproducible frame definition and hence document an approach for reliable interpretation of articulating joint movement patterns.

Our underlying hypothesis was that if discrepancies between kinematic datasets and the ground truth signals result from differences in frame alignment, then a set of frame rotations should exist, which, if applied to the segment frames, would compensate for misalignment and eliminate cross-talk errors. Such a method could ideally be applied independently to any given kinematic dataset, without requiring access to a full ground truth signal, and thereby provide a reliable and reproducible interpretation of the kinematic patterns for comparison across studies.

In order to describe the mathematical formulation, we initially provide the underlying notation:

Let rotation matrix ${\mathbf{R}}_{B}^{A}$ denote the orientation of frame A relative to frame B. ${\mathbf{R}}_{B}^{A}$ can be expressed in terms of *Tait-Bryan angles* as

1 $${\mathbf{R}}_{B}^{A}=\mathbf{R}\left(\widehat{\mathbf{x}}, \alpha \right)\times \mathbf{R}\left(\widehat{\mathbf{y}}, \beta \right)\times \mathbf{R}\left(\widehat{\mathbf{z}}, \gamma \right),$$

for an intrinsic XYZ sequence of rotation, where $\mathbf{R}\left(\widehat{\mathbf{v}}, \theta \right)$ indicates a positive rotation of $\theta$ around an axis in the direction of $\widehat{\mathbf{v}}$. Let us define, for convenience, ${\mathbf{r}}_{B}^{A}$ as the corresponding $3\times 1$ column vector, where the vector elements (in order from top to bottom) indicate e.g. knee joint flexion/extension, ab/adduction, and tibial int/external rotation,

2 $${\mathbf{r}}_{B}^{A}(t)=\left[\begin{array}{c}{{(\mathbf{r}}_{B}^{A})}_{1}\\ {{(\mathbf{r}}_{B}^{A})}_{2}\\ {{(\mathbf{r}}_{B}^{A})}_{3}\end{array} \right]=\left[\begin{array}{c}\alpha \\ \beta \\ \gamma \end{array}\right],$$

at timestep $$t$$.

**Figure 1:**
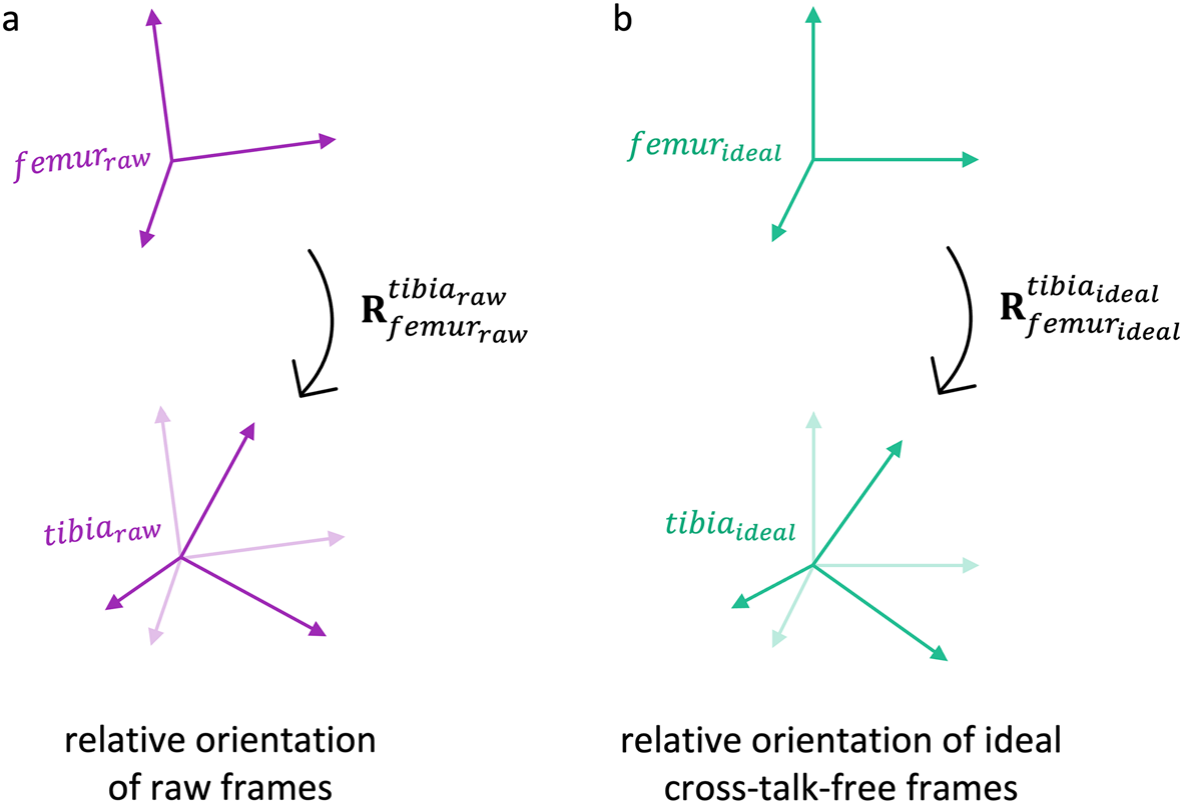
Schematic of local segment frames relative to one another: (**a**) Measured kinematics are given by rotation matrix ${{\varvec{R}}}_{{femur}_{raw}}^{{tibia}_{raw} },$ denoting the orientation of the raw tibial segment frame relative to the raw femoral segment frame. (**b**) A set of ideal kinematics would be given by rotation matrix ${{\varvec{R}}}_{{femur}_{ideal}}^{{tibia}_{ideal}}$, denoting the orientation of an ideal cross-talk-free tibial segment frame relative to an ideal cross-talk-free femoral frame.

Let us now consider a set of “raw” rotational knee kinematics, established based on measured data, expressed in matrix representation, where ${\mathbf{R}}_{{femur}_{raw}}^{{tibia}_{raw}}$ denotes the orientation of the tibial segment frame, ${tibia}_{raw}$, relative to the femoral segment frame, ${femur}_{raw},$ and varies with time (Fig. 1a).

The vector ${\mathbf{r}}_{{femur}_{raw}}^{{tibia}_{raw}}$ highly depends on both the order of the rotation sequence and the exact orientation of the individual femoral and tibial frames. Orientation of these frames is determined by the specific choice of approach used for coordinate system definition; be it landmark-based, functional, or a combination of the two. Any difference in frame orientation, even a very minor one, is known to lead to differences in kinematic patterns, even if the physical relative movement between the underlying joint segments is fundamentally the same^14^. This is especially problematic because for a given set of collected data, any and all estimated segment coordinate systems will be subject to some level of uncertainty or error.

**Figure 2:**
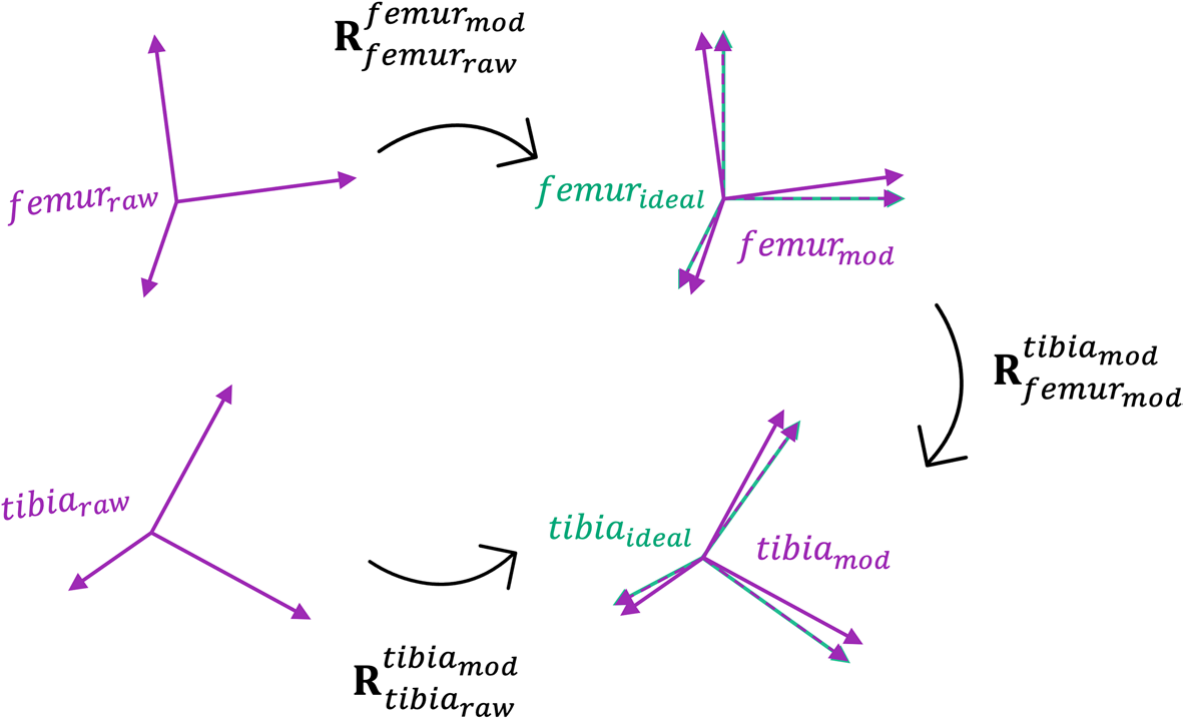
Schematic of raw (solid purple), ideal (solid green) and modified (dashed purple) local segment frames relative to one another, where modified frames are the raw frames after realignment to approximate the orientation of the ideal frames. ${{\varvec{R}}}_{{femur}_{raw}}^{{femur}_{mod}}$ and ${{\varvec{R}}}_{t{ibia}_{raw}}^{{tibia}_{mod}}$ denote the orientation of the modified frames relative to the orientation of the raw segment frames, and ${{\varvec{R}}}_{{femur}_{mod}}^{{tibia}_{mod}}$ denotes the orientation of the modified tibia frame relative to the modified femoral frame.

Consider an “ideal”, cross-talk-free kinematic signal, ${{\varvec{R}}}_{{femur}_{ideal}}^{{tibia}_{ideal}}$, resulting from the relative orientation of two frames, ${femur}_{ideal}$ and ${tibia}_{ideal}$ (Fig. 1b). At any given instant in time, it cannot be assumed that the raw frames are perfectly aligned with the orientation of these hypothetical ideal cross-talk-free frames (Supplementary Fig. S2). The difference in orientation between the raw and ideal frame of a given segment is constant over time, since both frames are defined as fixed relative to the (assumed to be) rigid bodies they represent. For measurements affected by soft-tissue artefact, an approach such as the Optical Common Shape Technique based on Procrustes approaches as described in^30^ would be required in order to ensure a rigid marker (or sensor) configuration. With sufficient knowledge of the raw frames’ and ideal frames’ respective orientations, it would then be possible to re-align the raw frames to match the ideal frames, hence allowing a set of “modified” or reorientated femoral and tibial frames, ${femur}_{mod}$ and ${tibia}_{mod}$ to be obtained (Fig. 2)*.* Let ${\mathbf{R}}_{{femur}_{raw}}^{{femur}_{mod}}$ and ${\mathbf{R}}_{t{ibia}_{raw}}^{{tibia}_{mod}}$ denote the orientation of the modified frames relative to the orientation of the raw segment frames. The relative orientation of these modified frames would yield kinematics given by rotation matrix ${\mathbf{R}}_{{femur}_{mod} }^{{tibia}_{mod}}$, where these modified kinematics are related to the raw kinematics by

3 $${\mathbf{R}}_{{femur}_{mod} }^{{tibia}_{mod} }= ({{\mathbf{R}}_{{femur}_{raw}}^{{femur}_{mod} })}^{-1}* {\mathbf{R}}_{{femur}_{raw}}^{{tibia}_{raw}}* \mathbf{R}_{{tibia}_{raw}}^{{tibia}_{mod} } .$$

Assuming the orientation of these ideal segment frames relative to the underlying segments are unknown, but the resulting relative rotations between the two segment frames, ${\mathbf{R}}_{{femur}_{ideal}}^{{tibia}_{ideal} }$ (or ${\mathbf{r}}_{{femur}_{ideal}}^{{tibia}_{ideal}}$), *are* known, then mathematical optimisation approaches could be used to solve for the values of ${\mathbf{R}}_{{femur}_{raw}}^{{femur}_{mod} }$ and ${\mathbf{R}}_{{tibia}_{raw}}^{{tibia}_{mod}}$ that minimise the differences between the modified, ${\mathbf{r}}_{{femur}_{mod}}^{{tibia}_{mod}}$, and the ideal, ${\mathbf{r}}_{{femur}_{ideal}}^{{tibia}_{ideal}}$, kinematics.

In most practical cases, knowledge of the numerical value of the hypothetical cross-talk-free ideal kinematics is admittedly not realistic. Although an undisputed definition of optimal segment frame orientations does not exist, partly due to a lack of consensus, but also due to differences in data capture approaches (including the consideration of soft-tissue artefact etc.), there is a common agreement that cross-talk artificially amplifies out-of-sagittal plane rotations (Supplementary Fig. S1). If absolutely no cross-talk were present, then pure joint flexion would not produce any artefact kinematic signal around the other axes. In a kinematic measurement, consisting of one dominant (e.g. flexion/extension) axis and two non-dominant (e.g. ab/adduction and int/external rotation) axes, the minimisation of rotations around the two non-dominant axes would inherently maximise rotation around the dominant axis, and therefore would not allow artefact rotations into the non-dominant axes. As a result, the remaining rotations in the non-dominant axes would not be distorted by cross-talk artefact. To achieve this, finding a frame alignment that is affected by as little cross-talk as possible can be enabled by determining the values of ${\mathbf{R}}_{{femur}_{raw}}^{{femur}_{mod} }$ and ${\mathbf{R}}_{{tibia}_{raw}}^{{tibia}_{mod}}$ that minimise ${({\mathbf{r}}_{{femur}_{mod}}^{{tibia}_{mod}})}_{2}$ and ${{(\mathbf{r}}_{{femur}_{mod}}^{{tibia}_{mod}})}_{3}$. While there are clearly various different cost functions that can be applied to minimise these values (each associated with different kinematic targets), for this demonstration of the approach, we have chosen to minimise root-mean-square (RMS). This choice implies that higher deviations from zero are weighted more heavily than if using the sum or average of the absolute values.

By re-aligning the segment frames to minimise the components of the 3D rotation that occur in the transverse and frontal planes, the magnitude of the rotation component in the third (sagittal) plane is thus effectively maximised. We therefore ensure that flexion predominates and that cross-talk is minimised. Furthermore, the need for a second associated kinematic dataset to act as the assumed ideal ground truth is eliminated. In practice, such a minimisation would be applied over the entire activity cycle. As such, it is not possible to entirely mitigate cross-talk errors for every instant of time. However, the overall output will indeed produce a consistent and reliable set of data, and hence allow comparison across trials, subjects, and studies.

### Application

As a first step towards approach verification, results of a previous investigation were used to explore the potential relationships between the magnitude of errors in different planes^13,31^. In a previous study, in vivo kinematics for six subjects over five valid cycles of three activities of daily living (level walking, stair descent, and sit-stand-sit) were derived from moving videofluoroscopy, using a cylindrical axis approach (i.e. based on the fitting of a cylindrical shape to each femoral condyle) to define the primary joint axis^31^. Mean kinematic signals for each subject were then replicated in a six-degrees-of-freedom robotic joint simulator (VIVO, AMTI, Watertown, MA) and measured using IMUs^13^. The ground truth data was then compared against IMU-based estimates obtained using an algorithm that leveraged the combined use of simple biomechanical models and Kalman smoothing^32^ to estimate functional axes and the associated knee joint angles from linear acceleration and angular velocity measurements. Although the simulator segment kinematics were consistent and unaffected by soft-tissue artefact, maximum absolute errors between the two kinematic datasets of up to 10.8° were observed. Larger errors in the transverse plane rotations seemingly coincided with higher flexion angles; a trend indicative of cross-talk between coordinate system axes (Supplementary Fig. S1).

In a preliminary frame orientation analysis, we first tested the assumption that the observed errors originated from cross-talk by applying mathematical optimisation (in this case, using a Levenberg–Marquardt algorithm^33^) to solve for ${\mathbf{R}}_{{femur}_{raw}}^{{femur}_{mod}}$ and ${\mathbf{R}}_{{tibia}_{raw}}^{{tibia}_{mod}}$ to minimise the RMSE between ${\mathbf{r}}_{{femur}_{mod}}^{{tibia}_{mod}}$ and ${\mathbf{r}}_{{femur}_{ideal}}^{{tibia}_{ideal}}$, (i.e. minimise $\sum_{i=1}^{3}\sqrt{\frac{1}{T}\sum_{t=0}^{T}{\left({\left({\mathbf{r}}_{{femur}_{ideal}}^{{tibia}_{ideal}}(t)\right)}_{i}-{\left({\mathbf{r}}_{{femur}_{mod}}^{{tibia}_{mod}}(t)\right)}_{i}\right)}^{2}}$) under the assumption that IMU-based estimates represented the raw data, and simulator ground truth represented the ideal data. Here, we used a specific implementation of FOOM to rotate the IMU-based reference frames to minimise differences between the IMU and simulator kinematic signals (i.e. FOOM_IMU→Sim_). By rotating the local femoral and tibial frames associated with the IMU data by ${\mathbf{R}}_{{femur}_{IMU}}^{{femur}_{mod}}$ and ${\mathbf{R}}_{{tibia}_{IMU}}^{{tibia}_{mod}}$, respectively, to align with the ground truth segment frames, a set of modified local frames was established and the resultant relative rotations were calculated. A comparison between the IMU kinematics resulting from these newly aligned frames and the simulator ground truth kinematics was then performed to establish how much of the reported errors were associated with cross-talk.

After this preliminary analysis established the level of cross-talk, the presented stand-alone implementation of FOOM to reorientate the segment reference frames was tested by comparing the IMU and simulator kinematic curves, which were derived from identical motion patterns but with different underlying reference frames. Here, each kinematic dataset was independently optimised by minimising the RMS of ab/adduction and int/external rotation (here, we minimised $\sum_{i=2}^{3}\sqrt{\frac{1}{T}\sum_{t=0}^{T}{{\left({\mathbf{r}}_{{femur}_{mod}}^{{tibia}_{mod}}(t)\right)}_{i}}^{2}}$); In contrast to FOOM_IMU→Sim_ in the preliminary analysis (which technically also involves optimising frame orientations to meet certain criteria), this latter broader implementation of FOOM individually considers both the simulator- and IMU-based kinematics as “raw” values in turn, acting as a self-contained approach that does not rely on information encompassed within a second dataset to achieve frame orientation optimisation.

Custom scripts to implement the described optimisations were developed in MATLAB (vR2021b; The Mathworks Inc., Natick, Massachusetts, USA). RMSEs were calculated for both the preliminary analysis (FOOM_IMU→Sim_) and the stand-alone implementation of FOOM. Paired t-tests were then conducted to compare RMSEs before and after frame reorientation, with and without a Bonferroni correction to account for multiple comparisons^34^ (assuming two independent comparisons were performed: 1—No Optimisation vs. FOOM_IMU→Sim_, and 2—No Optimisation vs. FOOM).

### Ethics declarations

This study used publicly accessible data and therefore did not directly involve humans. Collection of the original fluoroscopy data that was replicated here occurred within the scope of a separate cited study, which states that all subjects "provided written, informed consent to participate in this study, which was approved by the local ethics committee (EK 2011-N-6)"^31^.

## Results

### Preliminary analyses

**Figure 3:**
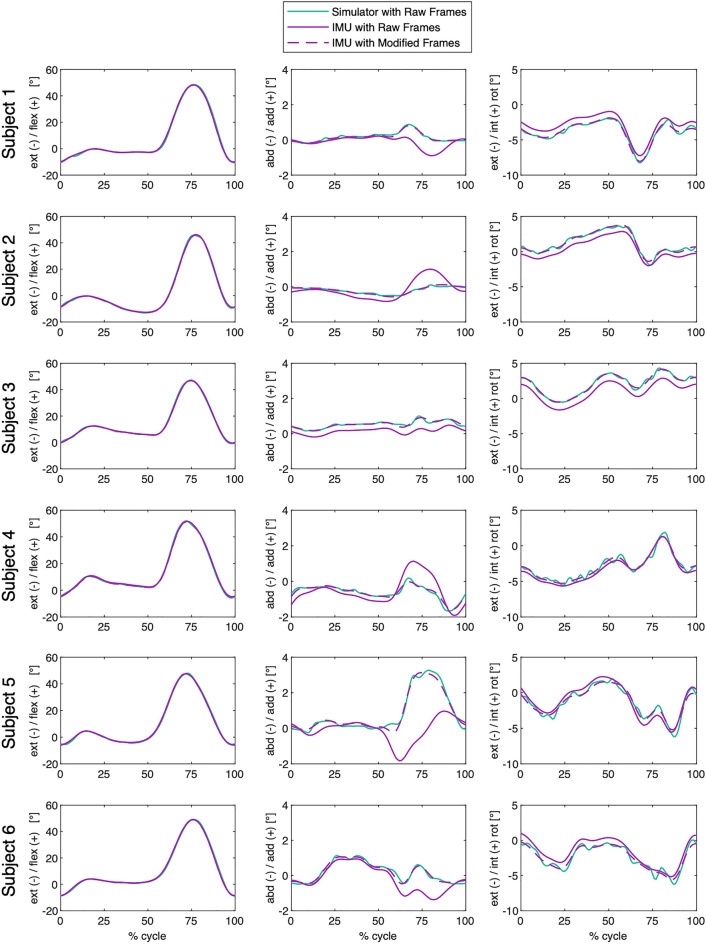
Level walking with raw frames: knee joint angles are shown over one complete exemplary gait cycle (expressed as a percentage) for each subject. The solid green lines illustrate the simulator kinematics, while the solid purple lines illustrate the IMU-based kinematics. The dashed purple lines show these IMU-based signals after rotation of the IMU- to the simulator reference frames (FOOM_IMU→Sim_), demonstrating convergence of the signals and a different interpretation of the movement patterns once aligned.

There were visible differences between the raw IMU-based and raw simulator kinematic patterns, despite identical underlying motion. The preliminary analyses (FOOM_IMU→Sim_) that aligned the IMU-based local segment frames to that of the simulator resulted in a clear convergence of the kinematic signals in all three planes, throughout the entire activity cycles and for all subjects—for brevity, only images for level walking are shown (Fig. 3), but figures for stair descent and sit-to-stand-to-sit can be viewed in the Supplementary Material (Figs. S3, S4), along with the corrective rotations applied to the femoral and tibial frames as part of these analyses (Tables S1, S2). Importantly, these improvements were associated with a considerable reduction in average RMSEs across all three activities for ab/adduction (from 0.7°–3.2° to 0.1°–0.5°) and for int/external rotation (from 0.8°–5.1° to 0.3°–0.6°) (Tables 1 and 2).

**Table 1:** RMSE ± standard deviation (in degrees) between the raw IMU-based kinematics and the raw simulator kinematics.

Not optimised	Flexion/extension	Ab/adduction	Int/ext rotation
Level walking	0.7 ± 0.1	0.7 ± 0.5	0.8 ± 0.2
Stair descent	0.7 ± 0.1	1.4 ± 1.1	1.4 ± 0.9
Sit-stand-sit	0.9 ± 0.3	3.2 ± 1.7	5.1 ± 2.1

**Table 2:** RMSE ± standard deviation (in degrees) between the IMU-based kinematics with rotated segment frames and the raw simulator kinematics (i.e. FOOM_IMU→Sim_).

Preliminary analyses	Flexion/extension	Ab/adduction	Int/ext rotation
Level walking	0.7 ± 0.1	0.1 ± 0.0	0.3 ± 0.1
Stair descent	0.6 ± 0.1	0.3 ± 0.2	0.4 ± 0.2
Sit-stand-sit	0.8 ± 0.2	0.5 ± 0.2	0.6 ± 0.2

### Frame orientation optimisation method

**Figure 4:**
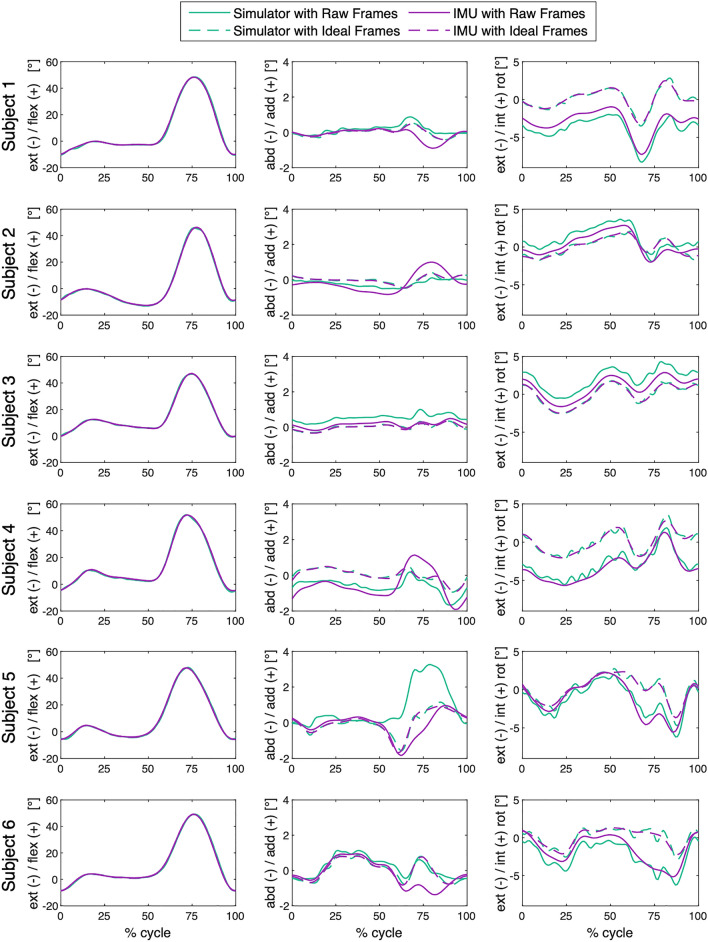
Level walking with ideal frames: Knee joint angles are shown over one complete exemplary gait cycle (expressed as a percentage) for each subject. The solid green lines illustrate the raw simulator kinematics, while the solid purple lines illustrate the raw IMU-based kinematics. The dashed purple lines show these IMU-based kinematics after frame orientation optimisation, while the dashed green lines show the simulator kinematics after optimisation, demonstrating convergence of the two sets of signals and a different interpretation of the movement patterns once aligned. Note that the converged signals differ from both original datasets but become consistent with one another.

After independent frame orientation optimisation of each raw dataset using the described FOOM, average RMSEs across all activities decreased to 0.1°–0.5° for ab/adduction and 0.3°–0.6° for int/external rotation (Fig. 4, Table 3). Figures for stair descent and sit-to-stand-to-sit can be viewed in the Supplementary Material (Figs. S5–S6), along with the corrective rotations applied to the femoral and tibial frames for all three activities (Tables S3–S6).

**Table 3:** RMSE ± standard deviation (in degrees) after application of FOOM to both the IMU- and simulator datasets.

Frame orientation optimisation method	Flexion/extension	Ab/adduction	Int/ext rotation
Level walking	0.7 ± 0.1	0.1 ± 0.0	0.3 ± 0.1
Stair descent	0.7 ± 0.2	0.3 ± 0.2	0.4 ± 0.2
Sit-stand-sit	1.0 ± 0.2	0.5 ± 0.2	0.6 ± 0.2

### Statistical analyses

**Figure 5:**
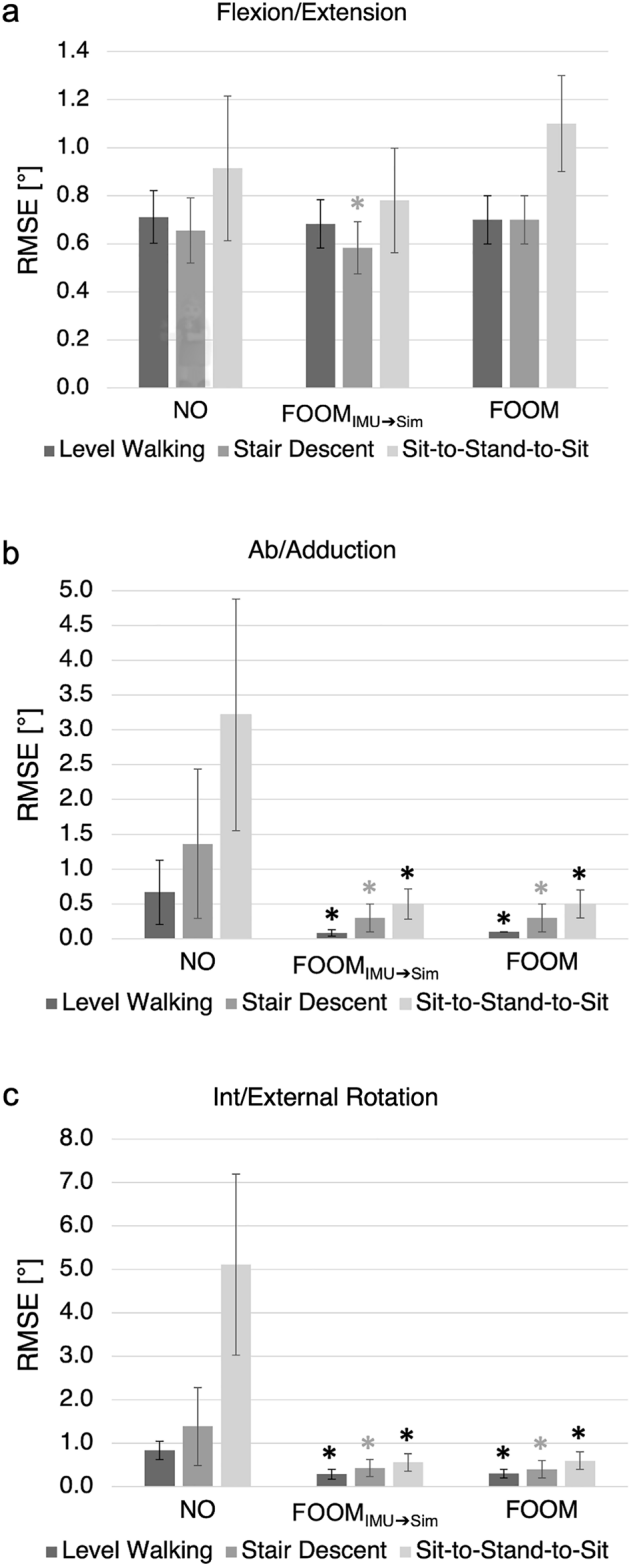
Root-mean-square error (RMSE) comparison: Average root-mean-square errors ± 1 standard deviation, before optimisation (*NO* — Not Optimised), after initial cross-talk analysis (FOOM_IMU→Sim_), and frame orientation optimisation based on minimisation of ab/adduction and int/external rotation RMS (FOOM), for all three activities: (**a**) flexion/extension, (**b**) ab/adduction, and (**c**) int/external rotation. Statistically significant differences based on a paired t-test with significance considered at 0.05 are indicated by an asterisk, where black asterisks indicate statistically significant differences after Bonferroni correction.

For the most part, neither the FOOM_IMU→Sim_ implementation in the preliminary analysis nor the stand-alone implementation of FOOM led to statistically significant changes in flexion/extension RMSE compared to the raw data (with the exception of FOOM_IMU→Sim_ analysis of stair descent, when Bonferroni correction was excluded; Fig. 5a). On the other hand, paired t-tests showed ab/adduction RMSEs to be significantly improved after frame optimisation for all activities (except stair descent if we perform a Bonferroni correction; Fig. 5b). Average RMSEs for ab/adduction decreased from a range of 0.7°–3.2° to 0.1°–0.5° after FOOM_IMU→Sim_ analysis, and to 0.1°–0.5° after full frame orientation optimisation (stand-alone FOOM). Similar outcomes were observed for int/external rotation RMSEs, which were significantly reduced for all activities (once again except stair descent if a Bonferroni correction is considered), from an average range of 0.8°–5.1° to 0.3°–0.6° (Fig. 5c).

## Discussion

In human movement science, the interpretation of joint motion around each axis is known to strongly depend upon the orientation of the chosen local coordinate frames. Due to variability in measurement and analysis approaches between institutions, the assessment of motion patterns remains insufficiently reliable to support clinical decision-making. In this study, we present the Frame Orientation Optimisation Method that has clearly demonstrated efficacy in unifying frame orientation to mitigate cross-talk in kinematic datasets, thereby providing a repeatable and standardised output, regardless of the analysis approach used. Application of FOOM to measured joint kinematics could therefore provide an approach for universal comparison of movement data.

In our study, we have been able to successfully realise the convergence of kinematic datasets to a reproducible signal using datasets from a previous study^13^. Here, the observed errors between IMU-based estimates and ground truth kinematics from a robotic joint simulator were thought to be indicative of cross-talk—a hypothesis that could be verified by solving for a set of compensatory 3D rotation parameters. The results of applying corrective rotations as part of a preliminary analysis (using FOOM_IMU→Sim_) clearly demonstrated that the differences could be almost entirely removed for all tested datasets (Fig. 3, Tables 1 and 2), hence providing strong evidence that almost all of the original errors did indeed stem from differences in frame orientation.

In in vivo settings, a set of ground truth values is almost never available, and movement scientists have therefore attempted to standardise clinical motion data in order to allow suitable comparison across studies^15,19,35–37^. In order to address this challenge, a fundamental assumption of our FOOM approach was that an ideal orientation exists for each of the segment frames. Consequently, instead of assuming that the ideal segment frames are prescribed by either ground truth data or the measured local segments’ anatomical or functional data (like other cross-talk reduction approaches^22,24^), our premise is that an alternative set of frames exists that is able to minimise cross-talk between axes. In our study, we demonstrated this postulation by minimising the RMS of ab/adduction and int/external rotation. Since it cannot be assumed that the simulator segment frames were defined to comply with the same criteria, an analogous transformation was applied to the simulator-based data (Fig. 4). These optimisations resulted in a third converged kinematic signal that could be consistently achieved from different kinematic datasets for the given motion patterns. It is important to note that implementation of the chosen criteria does *not* assume that ideal natural joint motion should consist of pure flexion/extension, nor that the resulting modified signal should approximate to a constant 0°; neither does it imply that the optimisation will actually produce that result. This application of FOOM should, however, maximise flexion/extension and therefore minimise the level of cross-talk artefact between axes.

Although the FOOM approach may redefine the motion planes, the method possesses the considerable advantage of being entirely self-contained; optimisation of a kinematic dataset based on segment frame alignment can be achieved without relying on information contained within a second dataset. While other approaches, such as the determination of functional joint axes^38^, also target the optimal orientation of the primary axis of rotation, our approach possesses the benefit of complete 3D frame re-orientation to minimise cross-talk around all axes. Naturally, different criteria might be better suited to optimise the alignment of segment frames during activities where flexion/extension does not clearly dominate, e.g. for a sidestep or crossover cutting manoeuvre. In such cases, out-of-sagittal plane rotations may themselves be of primary interest, and so minimisation of ab/adduction may not be appropriate.

Acknowledging that any optimisation-based method to standardise the representation of kinematic signals requires some flexibility, and offering this freedom to the user is a key difference between FOOM and methods such as those presented by Woltring^20^ or Rivest^23^. Accordingly, whether the implementation of a post-processing method like FOOM in fact leads to a *better* or *more accurate* set of kinematic data remains open for discussion. While finding consensus on the ideal definition of tibiofemoral kinematics is beyond the scope of this study, coordinate system definitions and alignment methods rely on one key assumption: that an optimal (ideal) alignment of the joint segment frames exists. However, *how* this alignment is defined and how it is best approximated based on the available data is ultimately a matter of *choice,* and further investigation towards standardising these choices is clearly required. While a certain choice of frame definition may be more (or less) suitable for answering a particular research question, the respective resultant kinematics cannot be considered to be more (or less) *accurate*, but rather simply a different (and hopefully more repeatable) representation of the same movement.

Here, the FOOM approach redefined the motion planes using rotational sequences, requiring only small angular corrections. In its current formulation, the algorithm could nevertheless find that larger rotations are needed to optimise the objective criteria for a different kinematic dataset. Rotating raw frames by larger angles to reach the desired modified frames does not hinder the underlying goal of determining whether differences in kinematic signals are caused by differences in frame orientation, rather than actual differences in the underlying movement patterns. If, however, the absolute values of the kinematic signals are believed to be clinically relevant, it is possible to ensure only small deviations from reference signals by modifying the underlying objective function to include a term that penalises deviations from one (or more) of the raw signals themselves. For example, it is possible to additionally minimise the difference between raw and ideal flexion values, or by constraining the magnitude of frame rotations permitted for optimisation.

For both the IMU-based data and the simulator values, kinematic calculations followed the globally recognised joint rotation convention of Grood and Suntay; a 3D Cartesian coordinate frame was attached to the femur and tibia, respectively, and angles were calculated as an intrinsic extension-adduction-internal rotation Cardan sequence of the tibia relative to the femur^15–17^. However, the simulator kinematic signals originally stemmed from values derived using a fluoroscopic dataset, where a cylindrical axis approach was used to define the femoral reference frame^31^. The IMU-based signals were derived with no direct information of the bone geometry, and therefore defined segment frames using a functional approach instead^13,32^. The converged signals of the optimised IMU and optimised simulator kinematics (Fig. 4) and the substantial reduction of ab/adduction and int/external rotation RMSEs after frame orientation optimisation (Fig. 5) indicate excellent agreement between these two datasets. This observed reduction in RMSEs after frame re-orientation suggests that the two dataset segment frames were not initially consistent with one another and were susceptible to cross-talk artefact, despite the fact they both represented the same underlying motion and followed the same Grood and Suntay rotation convention. It is therefore clear that comparable rotational kinematics require two key components: not only (1) a common joint rotation convention, but importantly also (2) common axis orientations in the local reference frames of the proximal and distal segments. While the former requirement is easily addressed within e.g. the Grood and Suntay convention, the latter is considerably more complicated and often completely ignored. As mentioned, the definition of axis orientations is generally approached geometrically based on the identification of anatomical landmarks, or functionally based on dynamic joint motion. However, although relationships between geometry and functional movement undoubtedly exist^39^, they are neither straightforward nor generalisable. The FOOM approach bypasses the need to relate differently defined axes of rotation by directly producing consistent and reliable kinematic signals.

In conclusion, our study has demonstrated that consideration of the exact orientation of reference frames, beyond basic conventional guidelines, is vital when drawing inferences regarding the (dis)agreement of two (or more) kinematic curves. Moreover, the optimisation of reference frames towards minimisation of cross-talk now allows a clear perspective for reliable comparison of kinematic data collected using different techniques and in different settings. As such, the presented approach provides new options for comparing e.g. IMU data, where the challenge of sensor-to-segment calibration has so far made valid comparisons difficult. Further investigation should clearly attempt to better understand what correct kinematics and optimally aligned joint frames entail, as well as further study methods of cross-talk quantification and their associated clinical applications^40^ and implications. Moreover, while the current examination was limited to rotational kinematics, a more comprehensive approach including translational kinematics should also be considered. By consistently standardising local segment frame alignment, such a collectively relevant approach will enable the valid comparison of kinematic data across trials, subjects, and studies.

## Supplementary Information

Supplementary Information.

## Data Availability

The datasets generated and analysed during the current study are available from the corresponding author on reasonable request.
