# Supplementary material for: A frame orientation optimisation method for consistent interpretation of kinematic signals
Source: Sci Rep. 2023 Jun 14;13:9632. doi: 10.1038/s41598-023-36625-z (PMC10267167; doi:10.1038/s41598-023-36625-z)
Supplement: Supplementary file 1 — Supplementary Information. [file 41598_2023_36625_MOESM1_ESM.pdf]

Supplementary Information: A Frame Orientation Optimisation Method for Consistent Interpretation of Kinematic Signals

Ariana Ortigas Vásquez*[1,2], William R. Taylor[3], Allan Maas[1,2], Matthias Woiczinski[2], Thomas M. Grupp[1,2], Adrian Sauer[1,2]

1. Research and Development, Aesculap AG, Tuttlingen, Germany;
2. Department of Orthopaedic and Trauma Surgery, Musculoskeletal University Center Munich (MUM), Campus Grosshadern, Ludwig Maximilians University Munich, Munich, Germany;
3. Laboratory for Movement Biomechanics, ETH Zurich, Switzerland.

Corresponding author: ariana.ortigas_vasquez@aesculap.de

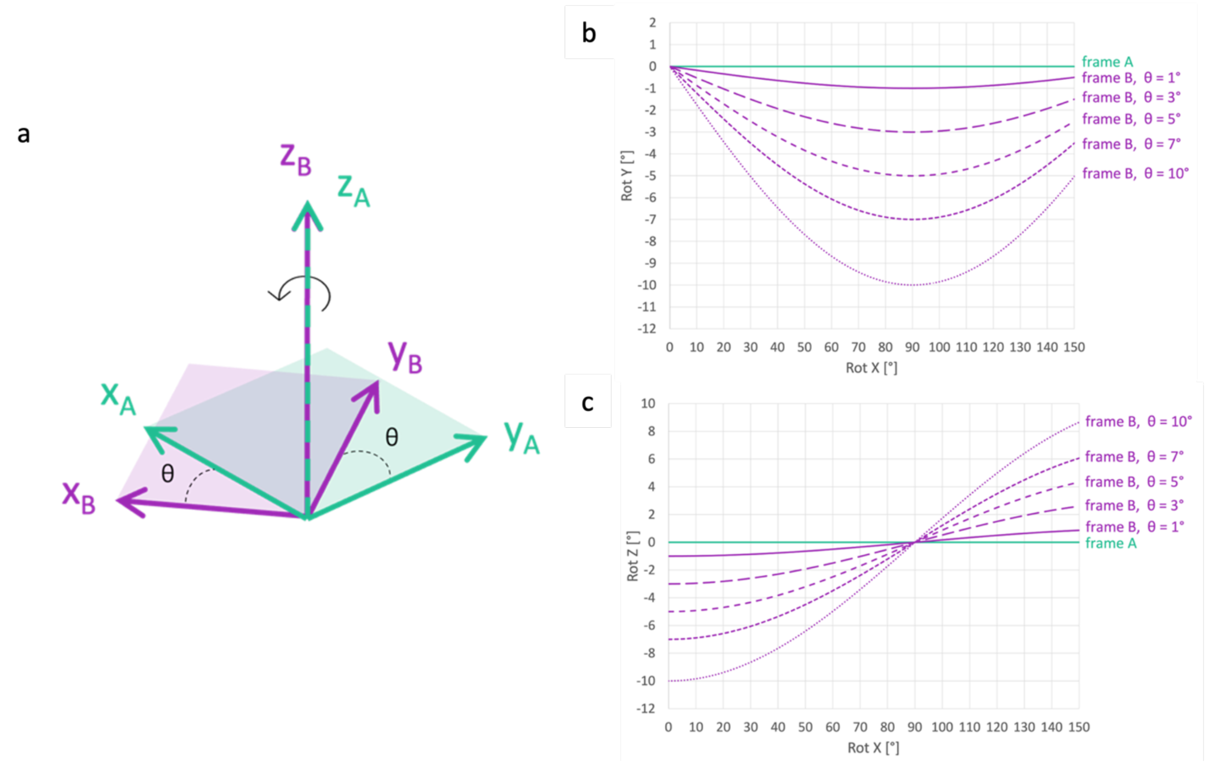

Supplementary Figure S1. Frame A (green) and Frame B (purple) are misaligned by a rotation of θ around the z-axis (a). A pure rotation around Frame A's x-axis is not perceived by Frame B as a pure rotation around its x-axis, but rather as rotations around its y-axis (b) and z-axis (c) as well.

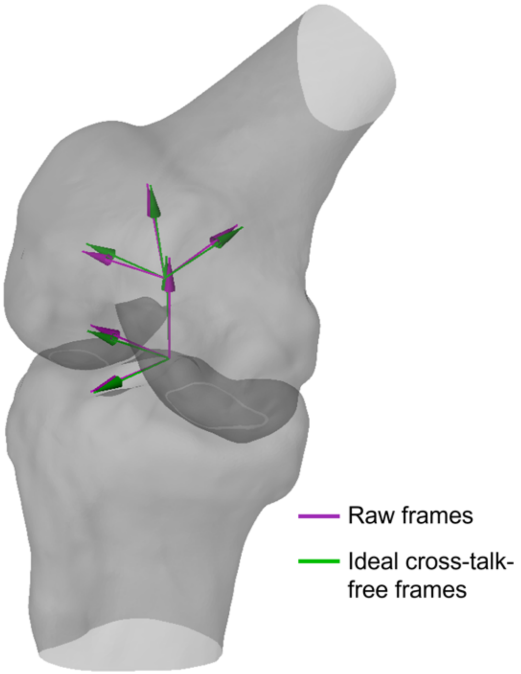

Supplementary Figure S2. Raw segment frames (purple) may not be perfectly aligned with the orientation of ideal cross-talk-free frames (green).

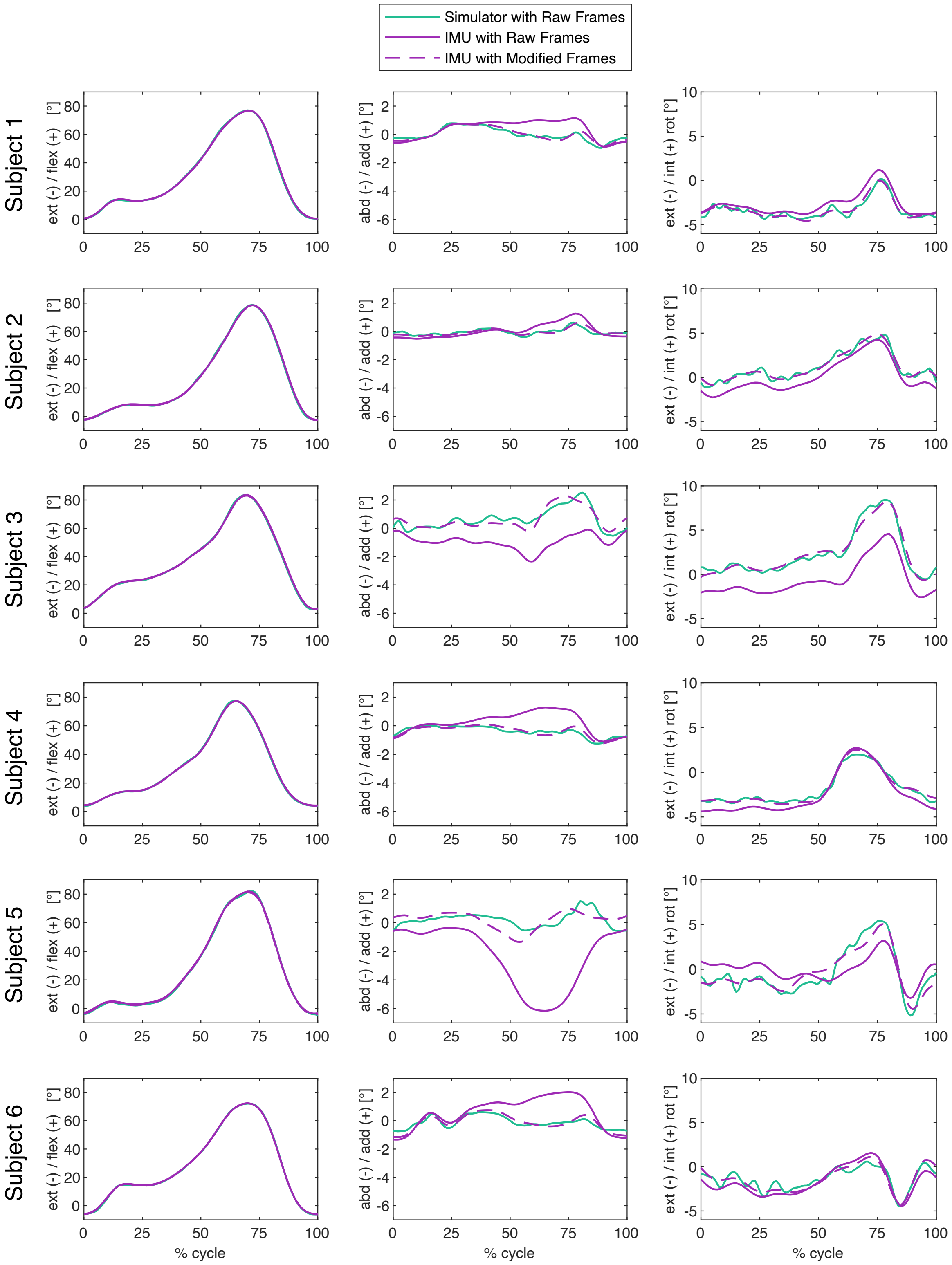

Supplementary Figure S3. Stair Descent ($FOOM_{IMU \rightarrow Sim}$): Knee joint angles are shown over one complete exemplary gait cycle (expressed as a percentage) for each subject. The solid green lines illustrate the simulator kinematics, while the solid purple lines illustrate the IMU-based kinematics. The dashed purple lines show these IMU-based signals after rotation of the IMU-to the simulator reference frames, demonstrating convergence of the signals and a different interpretation of the movement patterns once aligned.

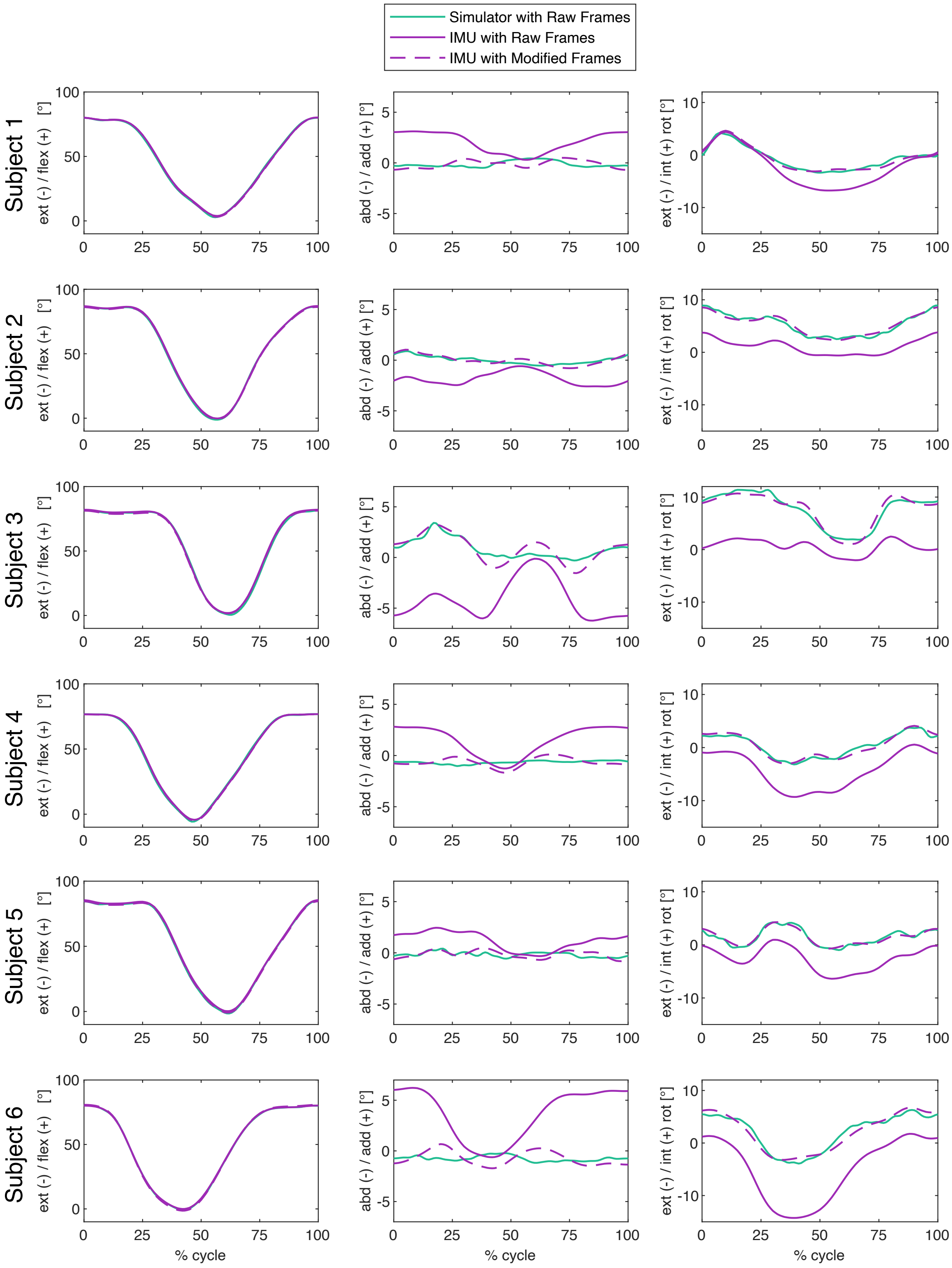

Supplementary Figure S4. Sit-to-Stand-to-Sit (FOOM$_{IMU \rightarrow Sim}$): Knee joint angles are shown over one complete exemplary gait cycle (expressed as a percentage) for each subject. The solid green lines illustrate the simulator kinematics, while the solid purple lines illustrate the IMU-based kinematics. The dashed purple lines show these IMU-based signals after rotation of the IMU- to the simulator reference frames, demonstrating convergence of the signals and a different interpretation of the movement patterns once aligned.

Supplementary Table S1. Corrective rotations in degrees applied on **femur** frame in preliminary analysis ($FOOM_{IMU \rightarrow Sim}$).

	Level Walking			Stair Descent			Sit-to-Stand-to-Sit		
	F/E	A/A	I/E	F/E	A/A	I/E	F/E	A/A	I/E
Subject 1	0.0	-0.5	1.0	0.1	1.5	-0.4	-0.1	3.8	0.3
Subject 2	0.2	0.7	-1.2	0.1	1.0	-0.3	0.3	-2.0	0.2
Subject 3	-0.1	-0.5	0.2	0.2	-2.4	0.1	0.7	-6.1	0.3
Subject 4	0.1	1.4	-1.4	-0.1	1.9	-0.4	-0.3	3.7	-0.3
Subject 5	-0.3	-3.3	3.3	0.5	-5.1	1.3	-2.3	2.4	0.5
Subject 6	-0.1	-1.5	1.1	0.1	2.2	-0.9	-2.0	6.7	0.0

Supplementary Table S2. Corrective rotations in degrees applied on **tibia** frame in preliminary analysis ($FOOM_{IMU \rightarrow Sim}$).

	Level Walking			Stair Descent			Sit-to-Stand-to-Sit		
	F/E	A/A	I/E	F/E	A/A	I/E	F/E	A/A	I/E
Subject 1	-0.1	-0.6	1.9	0.0	1.3	-0.3	-0.2	4.7	-3.9
Subject 2	0.0	0.7	-2.0	-0.3	0.8	-1.6	-0.2	-2.7	-2.8
Subject 3	-0.1	-0.8	-0.8	-0.1	-3.3	-1.5	0.2	-7.7	-2.5
Subject 4	-0.3	1.0	-2.0	-0.1	2.0	-1.8	-0.2	4.2	-7.1
Subject 5	-0.5	-3.5	3.8	-0.1	-6.1	3.3	-3.1	3.1	-5.4
Subject 6	-0.2	-1.6	2.1	-0.1	2.1	-2.0	-1.3	8.3	-11.4

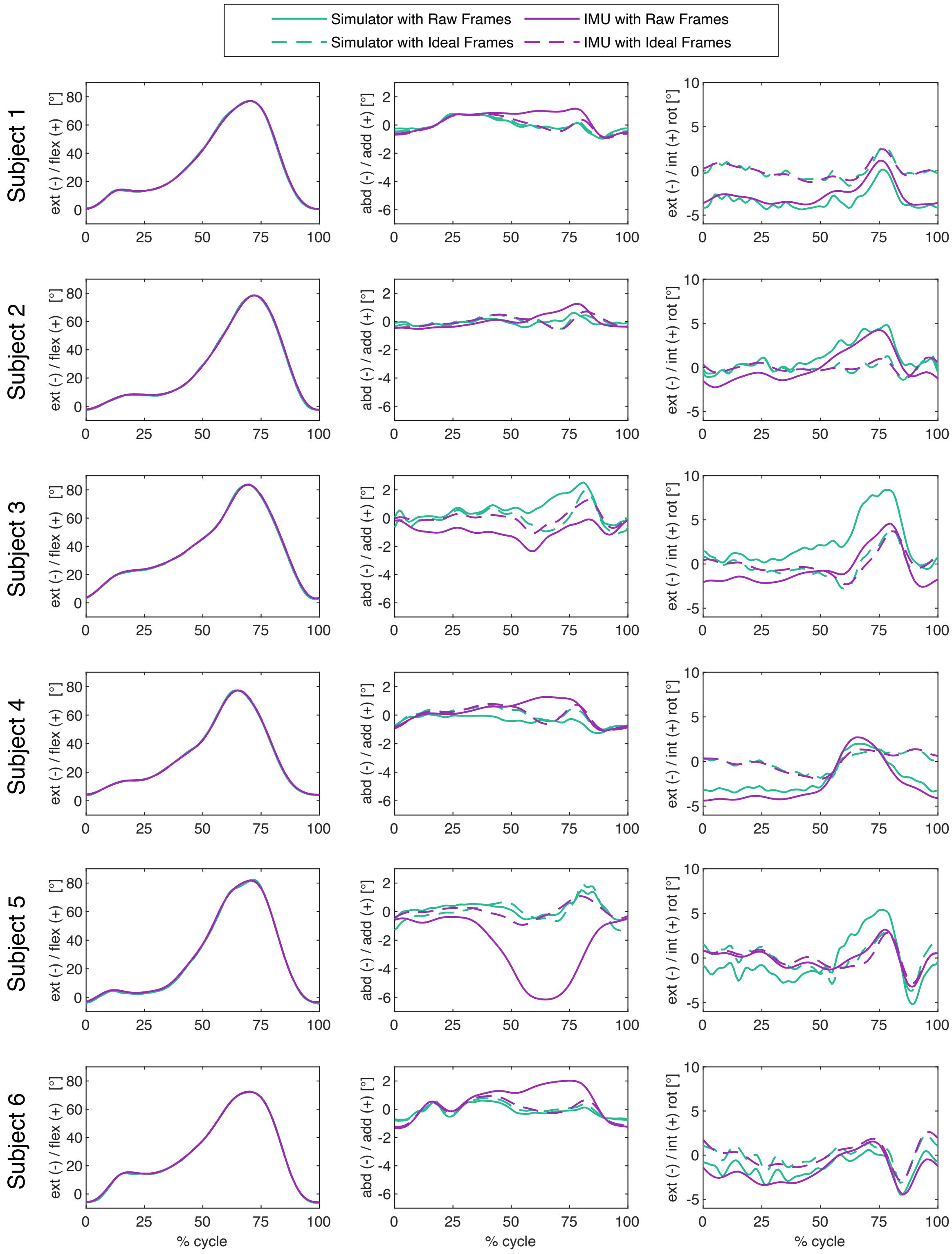

Supplementary Figure S5. Stair Descent (Frame Orientation Optimisation Method): Knee joint angles were plotted over the progression (expressed as a percentage) of one complete gait cycle. Purple lines illustrate inertially estimated angles, while green lines depict the joint simulator ground truth. Solid lines represent raw estimates, while dashed lines show kinematic values after frame orientation optimisation based on out-of-sagittal plane rotation RMSE minimisation. Each row represents one subject, and each column presents rotations in a different plane relative to the femoral coordinate system.

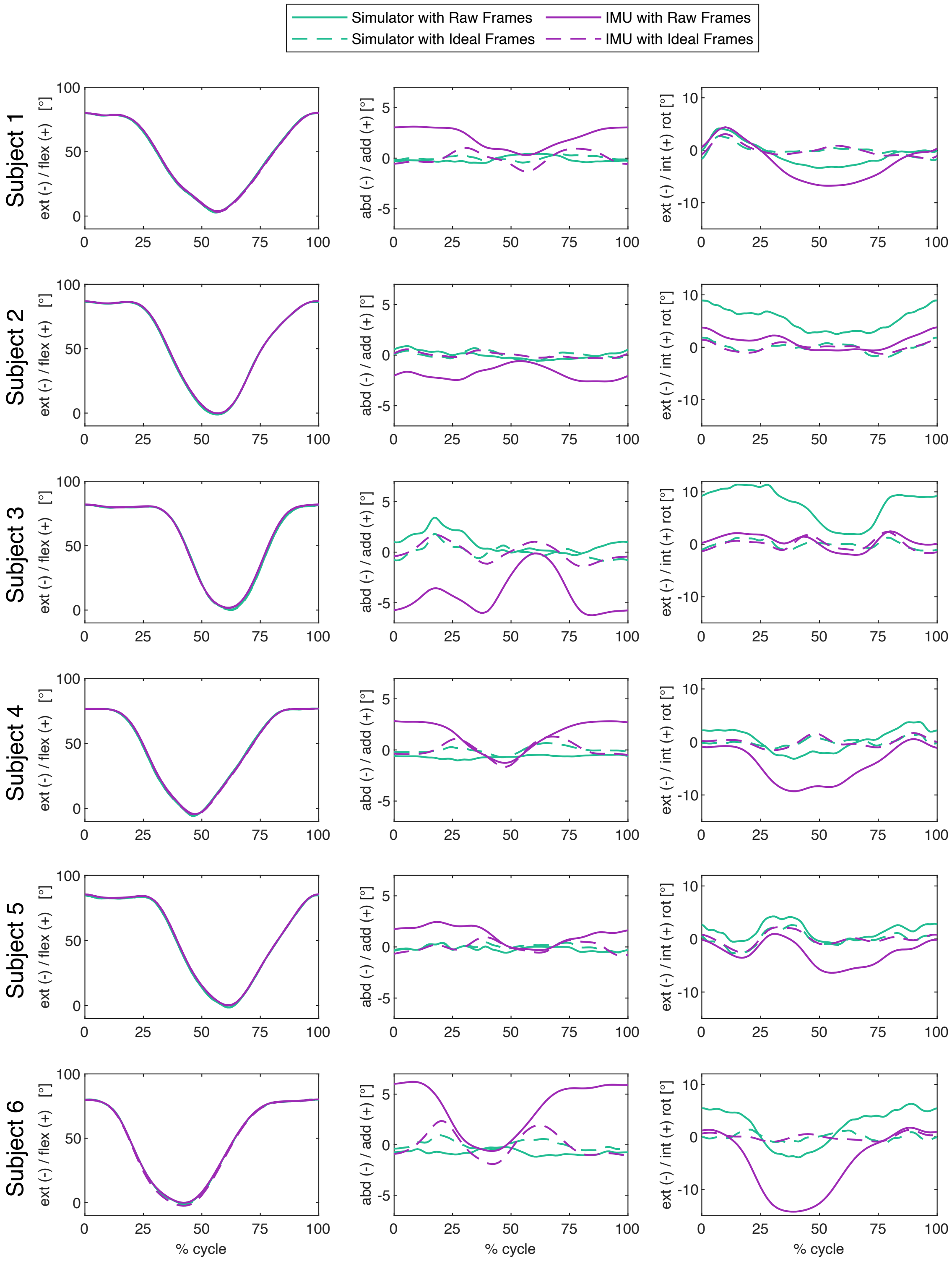

Supplementary Figure S6. Sit-to-Stand-to-Sit (Frame Orientation Optimisation Method): Knee joint angles were plotted over the progression (expressed as a percentage) of one complete gait cycle. Purple lines illustrate inertially estimated angles, while green lines depict the joint simulator ground truth. Solid lines represent raw estimates, while dashed lines show kinematic values after frame orientation optimisation based on out-of-sagittal plane rotation RMSE minimisation. Each row represents one subject, and each column presents rotations in a different plane relative to the femoral coordinate system.

Supplementary Table S3. Corrective rotations in degrees applied on **IMU femur** frame as part of frame orientation optimisation method (F/E = flexion/extension, A/A = ad/abduction, I/E = int/external tibial rotation).

	Level Walking			Stair Descent			Sit-to-Stand-to-Sit		
	F/E	A/A	I/E	F/E	A/A	I/E	F/E	A/A	I/E
Subject 1	0.1	-2.6	0.0	-0.1	2.4	0.6	-0.2	6.5	3.7
Subject 2	0.0	-2.3	-2.1	0.0	3.8	1.7	0.0	0.8	2.4
Subject 3	0.0	0.9	0.6	0.0	2.4	2.9	0.1	-0.7	3.7
Subject 4	-0.1	4.6	-0.5	-0.1	4.9	2.2	-0.2	6.7	2.3
Subject 5	0.1	-5.5	-0.6	0.1	-2.0	4.0	-0.1	3.7	1.3
Subject 6	0.1	-5.2	0.0	0.0	2.9	-0.3	-0.3	11.6	4.2

Supplementary Table S4. Corrective rotations in degrees applied on **IMU tibia** frame as part of frame orientation optimisation method (F/E = flexion/extension, A/A = ad/abduction, I/E = int/external tibial rotation).

	Level Walking			Stair Descent			Sit-to-Stand-to-Sit		
	F/E	A/A	I/E	F/E	A/A	I/E	F/E	A/A	I/E
Subject 1	-0.1	-2.6	-2.7	0.1	2.5	-3.3	0.2	8.3	-4.3
Subject 2	0.0	-2.5	-1.5	0.0	3.8	0.0	0.0	0.2	1.7
Subject 3	0.0	1.1	1.3	0.0	2.4	0.3	-0.1	-1.7	2.8
Subject 4	0.1	3.6	-4.8	0.1	5.1	-2.9	0.2	7.0	-7.2
Subject 5	0.0	-5.3	-0.8	-0.1	-2.4	3.9	0.1	4.0	-4.6
Subject 6	-0.1	-5.1	-0.7	0.0	2.8	-3.1	0.3	13.0	-10.2

Supplementary Table S5. Corrective rotations in degrees applied on **SIM femur** frame as part of frame orientation optimisation method (F/E = flexion/extension, A/A = ad/abduction, I/E = int/external tibial rotation).

	Level Walking			Stair Descent			Sit-to-Stand-to-Sit		
	F/E	A/A	I/E	F/E	A/A	I/E	F/E	A/A	I/E
Subject 1	0.1	-1.9	-0.9	-0.1	0.9	0.9	0.0	2.6	3.3
Subject 2	0.0	-3.0	-0.9	0.0	2.8	1.9	0.0	2.7	2.3
Subject 3	0.0	1.3	0.4	0.1	4.7	2.7	0.4	5.5	3.5
Subject 4	-0.1	3.1	0.8	-0.1	2.9	2.6	0.0	3.1	2.6
Subject 5	0.1	-2.2	-4.1	0.0	3.1	2.8	0.0	1.2	0.9
Subject 6	0.1	-3.9	-1.1	0.0	0.6	0.6	0.2	4.8	4.5

Supplementary Table S6. Corrective rotations in degrees applied on **SIM tibia** frame as part of frame orientation optimisation method (F/E = flexion/extension, A/A = ad/abduction, I/E = int/external tibial rotation).

	Level Walking			Stair Descent			Sit-to-Stand-to-Sit		
	F/E	A/A	I/E	F/E	A/A	I/E	F/E	A/A	I/E
Subject 1	-0.1	-1.8	-4.5	0.1	1.1	-3.0	0.0	3.6	-0.4
Subject 2	0.0	-3.1	0.4	0.0	3.0	1.6	0.0	2.9	4.5
Subject 3	0.0	1.8	2.1	-0.1	5.6	1.8	-0.4	6.0	5.4
Subject 4	0.1	2.4	-2.9	0.1	3.1	-1.1	0.0	2.9	0.0
Subject 5	-0.1	-1.9	-4.8	0.0	3.7	0.6	0.0	1.1	1.1
Subject 6	-0.1	-3.7	-2.8	0.0	0.6	-1.2	-0.2	4.9	1.5
